# Clinical impact of the accelerate PhenoTest® BC system on patients with gram-negative bacteremia and high risk of antimicrobial resistance: a prospective before-after implementation study

**DOI:** 10.1186/s12941-023-00619-6

**Published:** 2023-07-29

**Authors:** Tal Brosh-Nissimov, Anka Tzur, Daniel Grupel, Amos Cahan, Nir Ma’aravi, Maya Heled-Akiva, Hasan Jawamis, Hanna Leskes, Erez Barenboim, Nadav Sorek

**Affiliations:** 1 Samson Assuta Ashdod University Hospital, Harefua st. 7, Ashdod, 7747629 Israel; 2 Faculty of Health Sciences, Ben Gurion University in the Negev, Be’er Sheva, Israel

**Keywords:** Antimicrobial treatment, Antimicrobial susceptibility testing, Rapid identification, Rapid AST, Antibiotic stewardship, Gram negative bacteremia

## Abstract

**Background:**

The Accelerate PhenoTest® BC system (AXDX) is a novel assay for rapid bacterial identification and antimicrobial susceptibility (AST). We report an evaluation of its impact on treatment of patients with Gram-negative bacteremia (GNB) with a high risk of antimicrobial resistance (AMR).

**Methods:**

A prospective single-center evaluation before and after implementation of AXDX in addition to standard-of-care (SOC) microbiology and antimicrobial stewardship program (ASP). Patients with GNB reported during laboratory working hours and prespecified risk factors for AMR were included. The primary outcome was an ASP-oriented beneficial antimicrobial change, defined as either an escalation of an inappropriate empiric treatment or de-escalation of a broad-spectrum treatment of a susceptible organism. Main secondary outcomes were time to an appropriate treatment, antimicrobial treatment duration, length of stay (LOS) and mortality.

**Results:**

Included were 46 and 57 patients in the pre- and post-intervention periods, respectively. The median time to an AST-oriented beneficial change was 29.2 h vs. 49.6 h, respectively (p < 0.0001). There were no significant differences in the time to appropriate treatment, LOS or mortality. Antimicrobial treatment duration was longer during the intervention period (10 vs. 8 days, p = 0.007). AXDX failed to correctly identify pathogens in all 6 cases of polymicrobial bacteremia. In two cases patient care was potentially compromised due to inappropriate de-escalation.

**Conclusions:**

AXDX implementation resulted in a 20.4-hour shorter time to an ASP-oriented beneficial antimicrobial change. This should be weighed against the higher costs, the lack of other proven clinical benefits and the potential harm from mis-identification of polymicrobial bacteremias.

## Introduction

Gram-negative bacteremia (GNB) is associated with significant morbidity and mortality. Appropriate empiric antimicrobials can affect outcomes [1], but is becoming increasingly difficult with the rise in antimicrobial resistance.

Commonly used microbiology laboratory workflow for GNB takes 2–3 days [2–4] and includes blood culture incubation in automated systems, Gram-staining of positive cultures, subsequent subcultures, matrix-associated laser desorption/ionization time-of-flight (MALDI-TOF) spectrometry identification of bacteria and antimicrobial susceptibility testing (AST) using manual or automated techniques.

The Accelerate PhenoTest® BC system (AXDX, Accelerate Diagnostics, Tucson, AZ) provides a shorter turn-around time (TAT) for identification and AST (to approximately 2 and 7 h, respectively) by using morphokinetic cellular analysis. Previous studies have evaluated the analytical performance of AXDX [2–10] and the clinical impact of its implementation for bacteremias [11–14], and specifically for GNB [15–22].

In this study, we tested the effect of AXDX implementation in the laboratory workflow for GNB in selected patients with perceived high-risk for antimicrobial resistance (AMR), in addition to standard of care (SOC) microbiology techniques and an antimicrobial stewardship program (ASP). We hypothesized that early AST determination may have a beneficial impact on antimicrobial use.

## Materials and methods

### Design

To characterize the specific added value of AXDX on patient care, we conducted a single-center prospective study comparing the antimicrobial treatment of patients with GNB before and after its implementation. During both the pre- and post-intervention periods, the laboratory used push notifications to alert patients’ department and the infectious diseases (ID) consultant after Gram staining of a positive blood culture bottle, and upon identification of GNB. The consultant examined the patients’ medical records to determine their eligibility for the study. During the intervention period, input from AXDX was used in addition to standard-of-care (SOC) microbiology methods and the institutional antimicrobial stewardship program (ASP). Antimicrobial interventions were recorded during the first five days of treatment.

### Setting

A 300-bed teaching hospital servicing a population of ~ 400,000. An on-site microbiology laboratory operates daily, between 8:00–23:00, with shorter working hours on weekends and holidays. An ID consultant performs antimicrobial stewardship daily on weekdays and is available for on-demand consultations on weekends. Local antimicrobial treatment protocols have been published for common infectious diseases, but clinicians may choose not to follow them. Pre-authorization by an ID consultant is required for certain reserved broad-spectrum antimicrobials such as carbapenems, ceftazidime/avibactam, colistin and tigecycline. Due to a high prevalence of extended spectrum beta-lactamase producing Enterobacteriales (ESBL-E), local protocols encourage the use of aminoglycosides as first line empiric treatment for urinary tract infections.

### Inclusion criteria

Patients with GNB and a high-risk for antimicrobial resistant (AMR) bacteria, defined as the presence of any one or more of the following: Age > 70y; intensive care unit (ICU) admission; long-term care facility (LCTF) residence; immunodeficiency; or previous hospitalization or documented antimicrobial treatment in the past 6 months. Cases were eligible for inclusion only if bacteremia was reported on working days between 8:00–16:00, hence enabling AST results before 23:00.

### Exclusion criteria

We excluded patients who died before blood culture Gram stain; were not expected to survive > 24 h; had previous positive blood cultures during the same culprit infection; and those who were not hospitalized. As there were two AXDX modules were operating in the laboratory, cases were excluded if both modules were in use.

### Data Recording

Electronic health record clinical and demographic data were collected. The Charlson score was used to assess comorbidities, and Pitt bacteremia score to assess the patient’s condition on the day of blood culture collection. Appropriate treatment was defined when AST proved the organism to be susceptible in-vitro to the antimicrobial agent used; A beneficial ASP-oriented change was defined when either (a) an inappropriate treatment was changed into an appropriate treatment, or (b) if a broad-spectrum treatment regimen was de-escalated to a narrower but still appropriate one. The definitions of escalation and de-escalation are shown in Table S1. Changes is antimicrobial treatment were recorded at pre-﻿specified times: (1) empiric therapy before notification of bacteremia (2) after notification of bacteremia (3) after organism identification (4) after the AST report.

### Laboratory analysis

#### Routine analysis

Blood culture bottles were incubated in BACT/ALERT VIRTUO instrument (bioMérieux SA, France). Positive cultures were Gram-stained and plated on blood, chocolate, and MacConkey agar plates. Bacterial identification to the species level was performed by MALDI-TOF MS (bioMérieux SA, France), and by the Vitek 2 identification system (bioMérieux SA, France). AST was performed using the Vitek 2 (AST N395 and N308 cards; bioMérieux, SA, France), and results were interpreted according to Clinical and Laboratory Standards Institute (CLSI) guidelines.

During the intervention period, AXDX was used in addition to the routine assays, following Gram staining of a positive blood culture bolttle, as described previously [5]. We ran the Accelerate PhenoTest BC kit (Accelerate Diagnostics, USA) on the Accelerate Pheno system according to the manufacturer’s instructions. A report of the findings was automatically generated by Accelerate Diagnostics Host application.

The AXDX AST results were compared to the Vitek 2 result. Category agreement was defined according to Hombach et al. [23], and categorized as follows: Very major error (false susceptibility), major error (false resistance), or minor error (intermediate versus susceptible or resistant). The discrepancy rate was calculated by dividing the number of discrepancy instances by the number of susceptibility tests.

### Outcomes

The primary outcome was the time to an ASP-oriented beneficial change (see above). Secondary outcomes were the proportion of beneficial changes performed; time to appropriate antimicrobial treatment; in-hospital mortality; length of stay (LOS); antimicrobial treatment duration; 30-day re-admission rate; and 90-day *Clostridioides difficile* infection (CDI).

### Statistical analysis

Antimicrobial treatment times were calculated beginning with first administration, except if onset of treatment was before blood culture sampling, for which blood sampling time was determined as onset of treatment. Comparisons were done using Fisher’s exact test for categorical variables and Student’s t-test or Mann-Whitney test for continuous variables, as appropriate. Analyses were performed with Prism Graphpad 9.4.1.

## Results

**Fig. 1:**
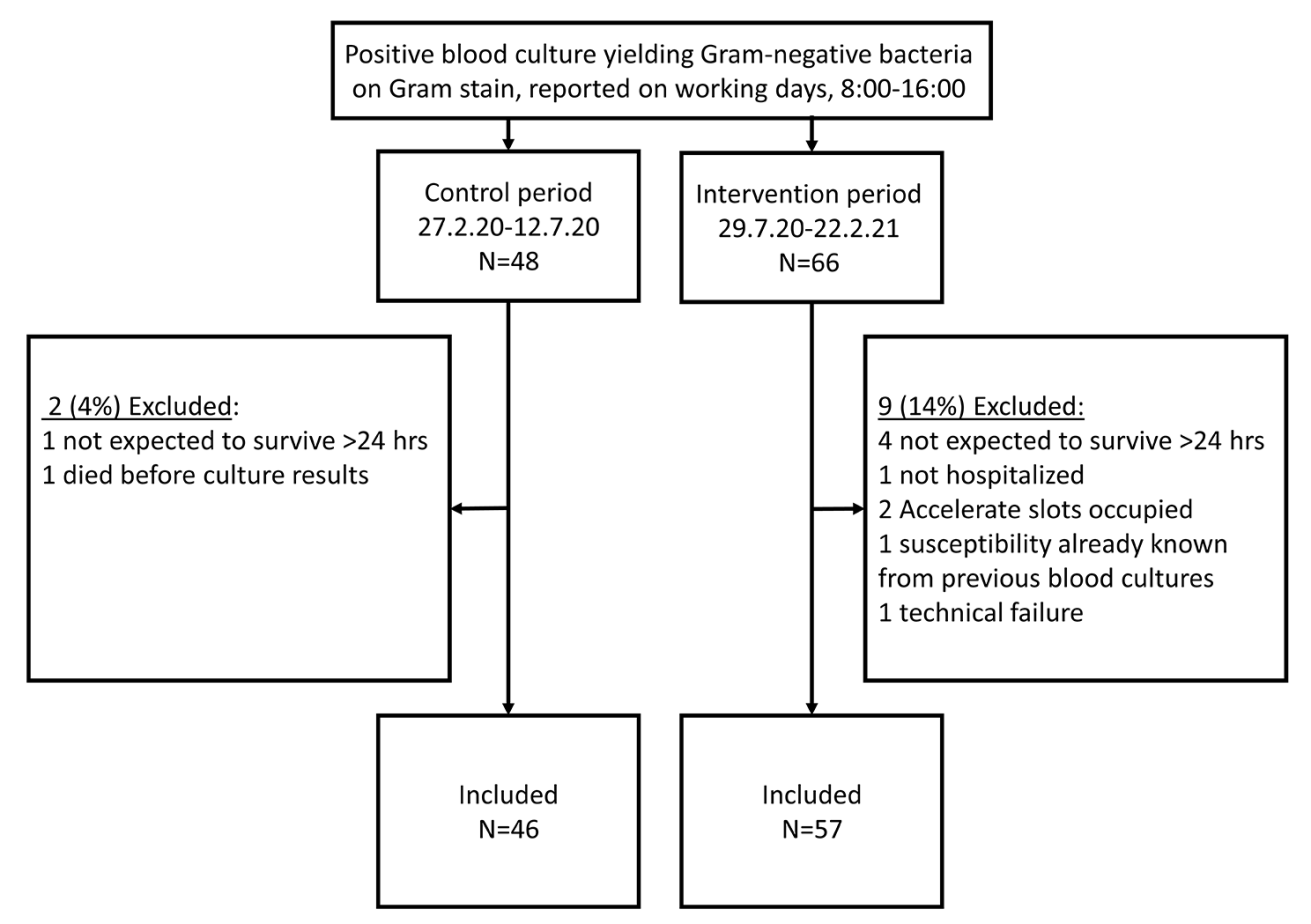
Flowchart of study population

During the pre- and post-intervention periods, 46 and 57 eligible patients with GNB, respectively, were prospectively followed (Fig. 1). There were no significant differences between the two patient groups in risk factors for AMR, Charlson comorbidity index, Pitt bacteremia scores, infection source, or microbiology of blood cultures (Table 1).

**Table 1:** comparison of patient groups in the pre-intervention and post-intervention study periods

			Control period	Intervention (AXDX) period	P value
N			46	57	
Time period			26.2.20–11.7.20	28.7.20–21.2.21	
Age, median (IQR)			79 (65–84)	75 (68–82)	0.28
Inclusion criteria	Age > 70, N (%)		32 (73%)	40 (70%)	0.83
	Previous hospitalization, N (%)		25 (54%)	37 (65%)	0.32
	Previous antimicrobial treatment, N (%)		23 (50%)	36 (63%)	0.23
	Long term care facility residence, N (%)		9 (20%)	14 (25%)	0.64
	Immunosuppression, N (%)		10 (22%)	6 (11%)	0.17
	Intensive care unit admission, N (%)		7 (15%)	7 (12%)	0.78
Charlson comorbidity index, median (IQR)			6 (5–8)	6 (4–8)	0.74
Pitt bacteremia score, median (IQR)			2 (0–4)	2 (0–4)	0.95
Infection source	Urinary		24 (52%)	29 (51%)	> 0.99
	Non urinary		22 (48%)	28 (49%)	
		Respiratory	4 (9%)	2 (4%)	
		Abdominal	10 (21%)	7 (12%)	
		Intravascular catheter	4 (9%)	7 (12%)	
		Surgical site	0 (0%)	1 (2%)	
		Other	3 (6%)	5 (9%)	
		Unknown	1 (2%)	6 (11%)	
Bacteria	Enterobacterales	Ceftriaxone sensitive	21/39	32/51	
		Ceftriaxone resistant	17/39	17/51	0.38 (vs. sensitive)
		Carbapenem resistant	1/39	2/51	> 0.99 (vs. non-CRE)
		E. coli	25	27	
		Klebsiella	7	14	
		Other Enterobacterales	9	10	
	Non-fermenters	Pseudomonas aeruginosa	3	5	
		Acinetobacter baumanii – carbapenem sensitive	0	2	
		Acinetobacter baumanii – carbapenem resistant	2	1	
	Other		1	3	
Polymicrobial bacteremia, N (%)			2 (4%)	6 (11%)	0.29

Abbreviations: AXDX, Accelerate PhenoTest® BC system; IQR, interquartile range

### AXDX Analytical performance

With the use of AXDX, the median time to identification and AST results was shorter by 8.8 and 16.8 h, respectively (Table 2). When comparing AST results between AXDX and the routine method, there were 18 (3.6%) minor errors, 2 (0.4%) major errors (ceftazidime and ciprofloxacin), and 2 (0.4%) very major errors (amikacin and trimethoprim/sulfamethoxazole). In addition, there were six (11%) episodes of polymicrobial bacteremia (all with growth of two organisms) in the intervention period, none of which were correctly identified by AXDX. In all those episodes, the primary Gram stain revealed only monomorphic Gram-negative rods. In four cases AXDX identified only one of two bacteria (*Proteus*, *Klebsiella*, and *Serratia*), and failed to identify the other (*Morganella*, *Pseudomonas aeruginosa*, carbapenem-resistant *Acinetobacter baumanii*, included in the AXDX panel, and *Staphylococcus hominis*, which was not included in the AXDX panel); in one case both bacteria were not included in the AXDX panel (*Bacteroides fragilis* and *Pseudomonas stutzeri*); and in one case *Klebsiella* spp. was identified by AXDX in one blood culture bottle, whereas another bottle not analyzed by AXDX grew *Enterobacter* spp.

**Table 2:** analytical performance of AXDX vs. routine laboratory methods

			Control period	Intervention (AXDX) period	P value
Microbial identification	Time to identification, hrs, median (IQR)		30.4 (23.2–36.5)	21.6 (17.6–25)	**< 0.0001**
	Failure to identify bi-microbial bacteremia	Assay failure^a^	NA	5	
		Laboratory procedural failure^b^	NA	1	
Antimicrobial susceptibility testing	Time to result		44.9 (38-48.2)	28.1 (23.9–31)	**< 0.0001**
	Minor error		NA	18 (3.6%)	
	Major error		NA	2 (0.4%)	
	Very major error		NA	2 (0.4%)	

^a^ Assay failure: AXDX detected only one of two organisms in the sample

^b^ Procedural failure: AXDX correctly detected the organism in the sample, but another blood culture bottle grew a different Gram-negative organism

Abbreviations: AXDX, Accelerate PhenoTest® BC system; IQR, interquartile range

### Clinical impact

Empiric antimicrobials were appropriate in 36 (78%) and 44 (77%) of patients in the pre-intervention and post-intervention period, respectively (Table 3). Antimicrobial changes that were performed after the reporting of GNB and before any other testing were appropriate in 40 (87%) and 48 (83%) of cases in the pre- and post-intervention periods, respectively. Following bacterial identification but before AST results, three antimicrobial treatment changes were performed in each study period. After AST reporting changes were performed in 25 (57%) and 28 (60%) of patients, respectively, of which 19 (76%) and 16 (57%) were de-escalations of broad-spectrum antimicrobials.

**Table 3:** clinical impact of microbiological analysis of blood cultures in the pre-intervention and post-intervention study periods

		Control period	Intervention (AXDX) period	P value
Antimicrobial stewardship-beneficial change	N (%)	29 (63%)	31 (54%)	0.83
	Time to change, hours, mean ± SD	49.6 ± 19.6	29.2 ± 13.3	**< 0.0001**
Time to appropriate antimicrobial treatment, hours, median (IQR)	Entire cohort	3.1 (0.4–21.7)	6.8 (0.9–24.5)	0.32
	Only cases with an inappropriate empiric treatment	36.1 (22.7–66.4) [N = 10]	28.5 (19.8–31.1) [N = 13]	0.23
Empiric antimicrobial treatment	Appropriate treatment, N (%)	36 (78%)	44 (77%)	> 0.99
	Time to treatment, hours, median (IQR)	1.3 (0-5.9)	1.7 (0-7.7)	0.73
After Gram-negative bacteremia reporting	Antimicrobial change, N (%)	20 (44%)	13 (23%)	**0.03**
	Time to change, hours, median (IQR)	22.1 (13.5–32.6)	21 (16.5–24.9)	0.89
	Appropriate treatment after change, N (%)	40 (87%)	48 (84%)	0.78
	Beneficial change, N (%)	8/20	8/13	0.3
After bacterial identification	Antimicrobial change, N (%)	3 (7%)	3 (5%)	> 0.99
	Time to change, hours, median (IQR)	35.1 (22.5–45.2)	21 (16.5–30.9)	0.2
	Appropriate treatment after change, N (%)	40 (87%)	52 (91%)	0.53
	Beneficial change, N (%)	2/3	3/3	> 0.99
After antimicrobial susceptibility results	Antimicrobial change, N (%)	25 (57%)	28 (60%)	0.83
	Time to change, hours, median (IQR)	53.8 (45.6–71.4)	31.1 (26.4–39.4)	**< 0.0001**
	Appropriate treatment after change, N (%)	46 (100%)	57 (100%)	> 0.99
	Beneficial change, N	25/28	22/28	0.47
Mortality, n (%)		12 (26%)	10 (18%)	0.34
Attributable mortality, n (%)		9 (21%)	9 (16%)	0.61
Length of stay, days, median (IQR)		8 (4–10)	7 (4-11.5)	0.92
Antimicrobial treatment duration, days, median (IQR)		8 (5–11)	10 (7–14)	**0.007**
Re-admission within 30 days, n (%)		8/34	7/47	0.39
* C. difficile* infection within 90 days, n (%)		0	0	NA

Abbreviations: AXDX, Accelerate PhenoTest® BC system; SD, standard deviation; IQR, interquartile range

**Fig. 2:**
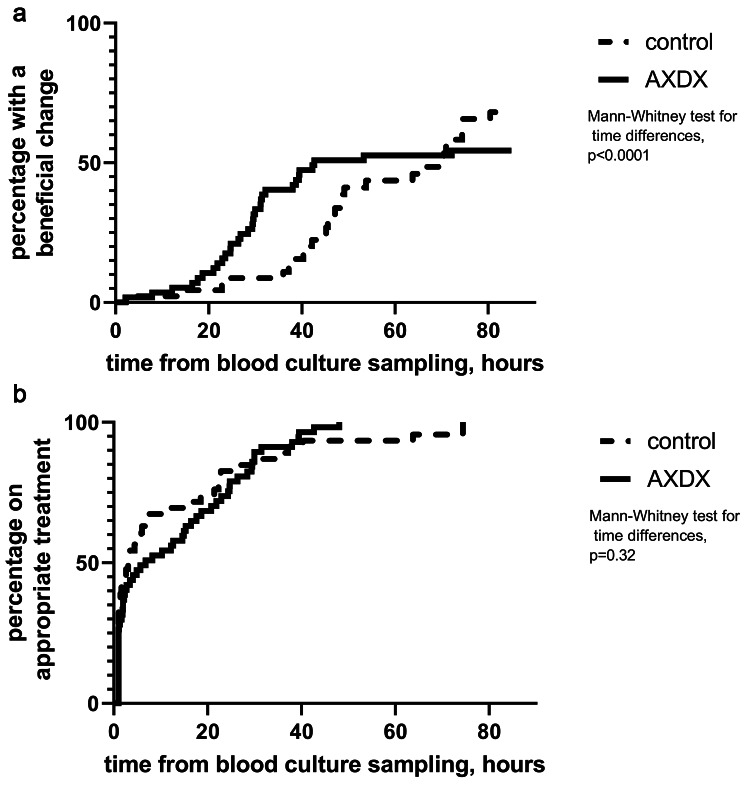
(**a**) Percentage of patients with a beneficial change according to time from blood culture sampling. (**b**) Percentage of patients receiving appropriate antimicrobials according to time from blood culture sampling

The primary outcome, i.e., time to an antimicrobial stewardship-oriented beneficial change, was significantly shorter in the intervention period. When comparing only cases with a beneficial change the median time to change was 29.2 h vs. 49.6 h, in the intervention vs. pre-intervention periods, respectively (Fig. 2a). The time to appropriate treatment was similar during the two periods, with a median of 3.1 and 6.8 h, respectively (Fig. 2b). The time to appropriate treatment was also similar in cases where empiric treatment was inappropriate. Median antimicrobial treatment duration was longer during the intervention period (10 vs. 8 days, p = 0.004). There were no differences between the study groups in other secondary outcomes, including mortality, LOS, re-admission rates or *C. difficile* infections.

None of the discrepancies in AXDX AST led to compromised patient care. Nevertheless, in cases of misidentification of polymicrobial bacteremias, a potential for compromised care was noted. In two cases of polymicrobial bacteremia, the treatment was inappropriately de-escalated according to the organism identified by AXDX, to an antimicrobial agent inappropriate for the other organism later identified by the microbiology laboratory (de-escalation from piperacillin/tazobactam to ceftriaxone in a *Klebsiella/P. aeruginosa* infection; and discontinuation of colistin from a regimen of colistin-meropenem in *Serratia/A. baumanii* infection).

## Discussion

We have implemented AXDX for rapid identification and AST, in a setting that included structured ID treatment protocols, active reporting of GNB to ID consultant and prospective audit and feedback. In a prospective evaluation of 46 and 57 patients with GNB and high risk for AMR, before and after the implementation of AXDX, respectively, we show a significant shorter time to a beneficial antimicrobial change, through escalation to an appropriate treatment, or, more commonly, de-escalation of a broad-spectrum treatment for a susceptible organism.

The merit of a novel rapid diagnostic method should be evaluated in various aspects, including analytical performance, the ability to shorten TAT in a meaningful way, and the impact on patient care.

The analytical performance of AXDX was evaluated in multiple studies. Correct microbial identification was obtained in 75.3–88.7% of bacteremias, and in 89.8–97.1% of bacteremias included on the system’s panel. AST results showed an overall category agreement of 92-97.9% and 92.7%-95.4% for GNB compared with culture-based AST [3–11, 15–17]. Our experience was similar with very few major and very major errors.

Nevertheless, AXDX performed poorly analyzing cultures with polymicrobial growth. None of six polymicrobial cultures (11% of cases) were correctly identified, either because the AXDX did not identify one of two organisms (5 cases) or because two culture bottles grew different organisms (1 case). None of these polymicrobial bacteremias were noticed at the primary Gram staining step, which was the trigger for AXDX performance. In 2 of these cases the incorrect identification by AXDX led to an inappropriate treatment de-escalation, potentially compromising patient care. We have not attempted to use AXDX for overt polymicrobial bacteremias, and so cannot estimate its performance for those. Nevertheless, AXDX cannot replace Gram staining of all positive culture bottles, and if performed on a single positive bottle, its results might not be representative of other bottles. Moreover, even for the single bottle that we have tested, polymicrobial bacteremias were still unidentified by the system. Low AXDX sensitivity in polymicrobial bacteremias was found in other studies. A study reported correct identification of all bacteria of mixed cultures in 12.5–40% of cases [2, 3], and in 68.8% when only bacteria included in the panel were considered [3]. In another study in which sterile site specimens were inoculated in blood culture bottles, 7/9 polymicrobial cultures were wrongly identified as monomicrobial [24]. In 6 studies investigating the clinical impact of AXDX implementation, 124 of 1444 cultures (8.6%) were found to be polymicrobial [11–13, 18–20]. In all these studies, polymicrobial cultures were excluded from the performance and impact analyses, although AXDX failure might have had a negative impact on patient care.

A significant shortening of TAT times was previously shown after AXDX implementation [2–5, 9–21]. In studies assessing AXDX clinical impact, the time to identification and AST was shorter than the SOC by 11.3–40.7 h [12–14, 18–21]. In our study the AST results time was only 16.8 h shorter, probably because the laboratory workflow supported performing and reading AST results during evening times.

Following the implementation of AXDX, the median time to an AST-oriented beneficial change was 20.4 h shorter. This finding is with agreement with other studies showing a decrease in the time to laboratory-guided antimicrobial changes and down-escalation of broad-spectrum antimicrobials in various clinical settings [11–14, 18, 19, 21, 22]. Of note, in all of these studies ASP was employed. In some studies [11, 18, 21], the time to appropriate treatment with AXDX was similar to that with SOC, as most patients received appropriate empiric treatment before culture results.

We did not find a difference between the study periods in robust clinical outcomes, including mortality, LOS, re-hospitalization or CDI incidence, and antimicrobial treatment duration was significantly longer during the intervention period. This is in agreement with a few previous studies that also did not show any impact on such outcomes [11, 12, 14, 19]. In contrary, Dare et al. showed a 1-day shorter LOS post positive culture identification (3 days for GNB), 1-day shorter duration of treatment and lower utilization of broad-spectrum antimicrobials [13]. Sheth et al. showed a 1-day shorter LOS and a shorter duration of broad spectrum treatment [22]. Bhalodi et al. reported a 1-day shorter LOS only for patients with GNB [14]. The most striking clinical impact was reported by Babowicz et al. with 85% lower risk of death after implementing AXDX [18]. As there was no difference between the intervention and control groups in the time to appropriate antimicrobial treatment, the reduced mortality could only be attributed to early treatment de-escalation. Indeed, unnecessary broad-spectrum empiric treatment was associated with a 1.22-fold higher risk of death in a large observational study [25]. Nevertheless, the reduced mortality reported in [18] seems to exceed the expected possible effect of antimicrobial de-escalation.

The impact of AXDX and other rapid AST technologies on patient care is complex. In order for an earlier AST result to lead to an earlier treatment change, clinicians need to be informed of the results, understand them, have confidence in the system and feel comfortable with de-escalation of treatment early on in a bacteremic patient course, when clinical signs of improvement may not yet be apparent [26]. In a study by Lee et al., in cases where AXDX revealed an inappropriate empiric treatment, escalation followed in most cases. In contrast, more than half of the opportunities to de-escalate treatment were missed [20].

ASP teams can facilitate antimicrobial changes in accordance with AST results based on AXDX analysis. However, early involvement of these teams in patient care might lead to a better initial appropriateness of empiric treatment, decreasing the potential benefit of rapid AST. For example, Walsh et al. reported shorter treatment duration and LOS after the implementation of the AXDX, however the intervention also included reporting of cases of bacteremia to the ASP team, which led to a significant increase in the involvement of ID consulting the management of bacteremic patients [21].

Rapid identification and AST using various laboratory methods were shown to increase cost effectiveness in the US healthcare system. None of these studies have included analyses of AXDX [27, 28]. Concurring with other studies, we also show the main benefit of AXDX in rapid antimicrobial de-escalation. Future studies are needed to determine whether AXDX is cost effective in terms of ASP. This study did not evaluate assay’s costs, or cost savings in antimicrobial treatment. Nevertheless, the additional costs of rapid assays are significant.

The strengths of this study include the similar use of laboratory reporting and ASP team involvement in patients’ care before and after AXDX implementation, which isolates the benefit of AXDX to its laboratory performance only; the inclusion of cases with a higher rate of AMR, who might have more benefit from rapid AST. In contrast to previous studies that excluded cases in which AXDX failed to identify bacteria (such as technical failures, incorrect identification of polymicrobial bacteremias etc.), we have included these cases, hence data was analyzed according to an “intention-to-diagnose” design. The study has some important limitations. As it was performed in a single and relatively small hospital, its results might not be generalizable to other settings, where larger volumes of cultures are processed, and early and intensive involvement of ASP clinicians in the management of bacteremic patients might not be feasible. The definitions of escalation and de-escalation are debatable in some cases, e.g., when switching between broad-spectrum antimicrobials such as third generation cephalosporins, quinolones, aminoglycosides etc. We have used the approach that directs ASP decisions in our institute. Last, due to the relatively small number of cases, our study was under-powered to detect differences in outcomes such as mortality and LOS.

## Conclusions

The implementation of AXDX for GNB with a high risk of AMR has benefited patient care by shortening the time to an antimicrobial change, mainly de-escalation of broad-spectrum antimicrobials. The earlier change, which was less than 24 h, should be weighed against the higher costs of AXDX, the lack of other significant clinical benefits, and the potential harm from consistent mis-identification of polymicrobial bacteremias.

## Data Availability

The datasets generated during and/or analysed during the current study are not publicly available due to patient privacy but are available from the corresponding author on reasonable request.
